# Hepatocellular carcinoma: clinicopathological profile and challenges of management in a resource-limited setting

**DOI:** 10.1186/1477-7819-12-246

**Published:** 2014-08-02

**Authors:** Hyasinta Jaka, Stephen E Mshana, Peter F Rambau, Nestory Masalu, Phillipo L Chalya, Samuel E Kalluvya

**Affiliations:** 1 Department of Internal Medicine, Catholic University of Health and Allied Sciences- Bugando, P.O. Box 1464, Mwanza, Tanzania; 2 Department of Microbiology & Immunology, Catholic University of Health and Allied Sciences- Bugando, P.O. Box 1464, Mwanza, Tanzania; 3 Department of Pathology, Catholic University of Health and Allied Sciences- Bugando, Bugando, P.O. Box 1464, Mwanza, Tanzania; 4 Department of Oncology, Catholic University of Health and Allied Sciences- Bugando, Bugando, P.O. Box 1464, Mwanza, Tanzania; 5 Department of Surgery, Catholic University of Health and Allied Sciences- Bugando, Bugando, P.O. Box 1464, Mwanza, Tanzania

**Keywords:** Hepatocellular carcinoma, Clinicopathological, Challenges, Resource-limited setting, Tanzania

## Abstract

**Background:**

Hepatocellular carcinoma is one of the most common cancers worldwide and its incidence is reported to be increasing in resource-limited countries. There is a paucity of published data regarding hepatocellular carcinoma in Tanzania, and the study area in particular. This study describes the clinicopathological profile of hepatocellular carcinoma in our local setting and highlights the challenging problems in the management of this disease.

**Methods:**

This was a retrospective study of histopathologically confirmed cases of hepatocellular carcinoma seen at Bugando Medical Center between March 2009 and February 2013.

**Results:**

A total of 142 patients (M: F = 2.2: 1) were studied representing 4.6% of all malignancies. The median age of patients was 45 years. Hepatitis B virus infection (66.2%) and heavy alcohol consumption (60.6%) were the most frequently identified risk factors for hepatocellular carcinoma. The majority of patients (88.0%) presented late with advanced stages. HBsAg was positive in 66.2% of the patients and Hepatitis C Virus antibody in 16.9%. Thirteen (9.2%) patients tested positive for HIV infection. Most patients (52.8%) had both right and left lobe involvement. The trabecular pattern (47.9%) was the most frequent histopathological type. None of patients had curative therapy because of the advanced nature of the disease. Coagulopathy (45.7%) was the most common complications. The overall mortality rate was 46.5% and it was significantly associated with comorbidity, HIV positivity, CD4+ count <200 cells/μl, high histological grade, advanced stage of the tumor, presence of distant metastases at the time of diagnosis, and associated complications (*P* < 0.001). The overall median duration of hospital stay was 14 days. The majority of patients (71.1%) were lost to follow-up at the end of the follow-up period.

**Conclusions:**

Hepatocellular carcinoma patients in this region are relatively young at diagnosis and the majority of them present late with an advanced stage and high rate of distant metastasis. Lack of awareness of the disease, poor accessibility to healthcare facilities, and lack of screening programs in this region may contribute to advanced disease at the time of diagnosis. There is a need for early detection, adequate treatment, and proper follow-up to improve treatment outcome.

## Background

Hepatocellular carcinoma (HCC) is one of the most common cancers worldwide with approximately 550,000 to 600,000 new HCC cases globally each year [1]. HCC is the third leading cause of cancer deaths worldwide, with a prevalence 16 to 32 times higher in developing countries than in developed countries [1,2]. This cancer has a heterogeneous geographical distribution based on the prevalence of risk factors in different parts of the world. It is frequent in Sub-Saharan Africa and southeast Asia where it is responsible for a large proportion of cancer deaths, but is rare in the United States and Europe [3,4]. The high incidence rate may be related to the high prevalence of Hepatitis B viral (HBV) infection, aflatoxin B1 contamination, and some hepatotoxic drugs [5]. Hepatitis C virus (HCV), cigarette smoking, and alcohol are also etiologic agents in this environment [6]. Recently, there is an upsurge of HCC in the United States due to increased HCV infection [7].

The transformation of hepatocytes to the malignant phenotype may occur irrespective of the etiological agents. In the context of increased cellular turnover induced by chronic liver injury and regeneration, the activation of cellular oncogenes or the inactivation of tumor suppressor genes are the common denominators contributing to the development of HCC [8]. The oncogenic mechanism of HBV is thought to be based on genetic damage associated with chronic inflammation and the integration of HBV DNA into the host genome [9]. Aflatoxin B1 has been shown to induce mutations at codon 249 of the *P53* tumor suppressor gene, thus providing a clue to how an environmental factor may contribute to tumor development at a molecular level [8,10]. HCC is an asymptomatic and slow-growing malignancy whose natural history is an extension of underlying cirrhosis [9]. This tumor is aggressive in black people and associated with poor prognosis [11,12].

HCC in black Africans carries a particularly grave prognosis, with average survival times from the onset of symptoms being as short as 14 weeks [2,13] and, with very few exceptions, all of the patients surviving for less than one year. The great majority of the population lives in rural areas where the incidence of the tumor is higher than it is in urban areas and where facilities for diagnosing and treating HCC are least adequate. HCC often occurs at a relatively young age in black Africans, and this is even more evident in those born and growing up in rural areas. Men are affected far more often than women [13].

The occurrence of HCC at such a high incidence in resource-limited countries and the advanced stage of the disease when the patients usually seek medical attention, as well as the inadequate diagnostic and, more importantly, treatment facilities for the tumor, pose an enormous challenge in managing HCC in these countries [2,11-13]. Other major challenges in the longer term management of HCC in developing countries are pre-symptomatic detection of the tumor and prevention of hepatitis virus infections, dietary exposure to aflatoxin B1, and dietary iron overload - the major causes of HCC in developing countries [2,13].

The clinical stage of the disease at diagnosis often determines the prognosis and survival rate of a patient with HCC, with the best outcomes seen in patients diagnosed at an early stage [2,12,13]. However, the outcome of treatment of HCC in our environment has been poor because the majority of these patients present late to the hospital with an advanced stage of the disease and only palliative care is possible [11,12]. This is partly due to a lack of community awareness on the importance of early reporting to hospital for the early diagnosis and treatment of this condition.

The prognosis of HCC in Sub-Saharan Africa is generally poor with patients usually presenting late with an advanced stage of the disease [11-13]. This is in contrast to what occurs in Western countries where the disease is increasingly being diagnosed at an early stage (when it is amenable to treatment), though regular screening of those at risk [14].

HCC screening programs have been reported to increase the detection of tumors at earlier stages and reduce incidence and mortality related to HCC [13,14]. In resource-limited countries, however, lack of a screening program in high-risk individuals poses a great challenge in the prevention of HCC.

There is a paucity of information regarding hepatocellular carcinoma in Tanzania and the study area in particular. This is partly due to a lack of published local data regarding this condition and the lack of cancer registries in this region. This study was designed to describe the clinicopathological pattern of HCC and highlight the challenging problem in the management of this disease in our local setting.

## Methods

### Study design and setting

This was a five-year retrospective study of histologically confirmed cases of hepatocellular carcinoma which was conducted at Bugando Medical Center between April 2008 and May 2013. Bugando Medical Center is a consultant, referral, and teaching hospital for the Catholic University of Health and Allied Sciences-Bugando (CUHAS-Bugando) in the Lake and Western zones of the United Republic of Tanzania. It is situated along the shores of Lake Victoria in Mwanza City. It has 1000 beds and serves as a referral center for tertiary specialist care for a catchment population of approximately 13 million people. The hospital has a newly established oncology department which provides care for all patients with histopathologically proven cancers, including hepatocellular carcinoma.

### Study population

The study population included all patients who presented to Bugando Medical Center with histologically confirmed hepatocellular carcinoma during the study period. Patients with incomplete data were excluded from the study. The details of patients were obtained from patients’ files kept in the medical record department, the medical and surgical wards, operating theatre, and histopathology laboratory. Information collected included sociodemographic data, clinical presentation, risk factors for HCC, investigations, anatomical site, macroscopic appearance, tumor stage, histopathological type and grade, presence of distant metastasis, treatment modalities, and outcome and follow-up.

Information on risk factors for chronic liver disease and HCC such as blood transfusion, previous jaundice, scarification mark and alcohol consumption, were obtained using a structured questionnaire. ‘Heavy alcohol intake’ was defined as a daily minimum consumption of 160 g alcohol for at least eight years, while cigarette smoking was defined as 10 sticks per day for at least 10 years. Regular intake of native herbal preparations for more than 10 years was considered herbal abuse. Diagnosis of HBV infection was based on positive serology for HBsAg using a microparticle enzyme immunoassay (MEIA) (Abbott Laboratories, AXSYM, United States). Diagnosis of HCV infection was based on positivity for anti-HCV antibodies, as determined by third-generation enzyme immunoassay (Abbott Laboratories). HIV testing was performed using the Tanzania HIV Rapid Test Algorithm [15] and CD4+ count using FACS or FACSCALIBUR (BD Biosciences, United States). A determination of CD4+ count was only performed in HIV-positive patients. HCC was diagnosed based on a histopathological examination. Cirrhosis was diagnosed histologically in most cases. We could not estimate the Alpha fetoprotein (AFP) levels or carry out computed tomography (CT)scan on the patients due to the lack of this equipment at our centre. The clinical stage of the disease was assigned to each patient using the Okuda classification [16].

### Statistical data analysis

The statistical analysis was performed using the Statistical Package for Social Sciences (SPSS) version 17.0 for Windows (SPSS, Chicago, Illinois, United States). The median (and IQR) and ranges were calculated for continuous variables, whereas proportions and frequency tables were used to summarize categorical variables. The chi-square (χ2) test was used to test for the significance of association between the independent (predictor) and dependent (outcome) variables in the categorical variables. The level of significance was considered as *P* < 0.05. Multivariate logistic regression analysis was used to determine predictor variables that predicted the outcome.

### Ethical consideration

Ethical approval to conduct the study was obtained from the CUHAS-Bugando/BMC joint institutional ethic review committee before the commencement of the study.

## Results

### Sociodemographic data

**Figure 1:**
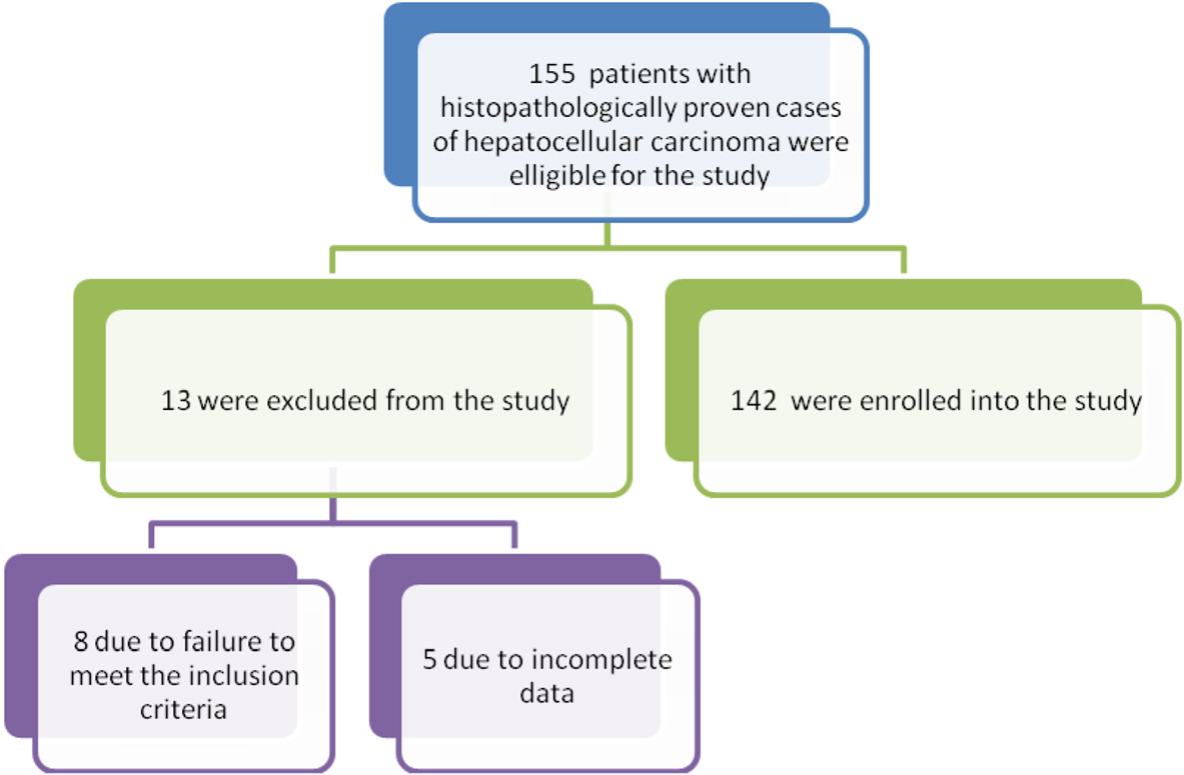
Flow chart of patients.

Out of 3104 patients who were registered with malignancies during the study period, 155 were histopathologically confirmed cases of hepatocellular carcinoma. Of these, 13 patients were excluded from the study due to incomplete data. Thus, 142 patients representing 4.6% of cases were enrolled into the study and this formed the study population (Figure 1). Of these, 98 (69.0%) were males and 44 (31.0%) were females with a male to female ratio of 2.2: 1. The age of patients at diagnosis ranged from 14 to 76 years with a median age of 45 years and (IQR of 38 to 53 years). The modal age group was 41 to 50 years, accounting for 47.2% of cases (Table 1). Patients with HBV-associated HCC presented at a younger age than those with HCV-associated HCC. This association was statistically significant (*P* = 0.004). The majority of patients (122, 85.9%) were farmers coming from rural areas located a considerable distance from the study area and more than 80% of them were unemployed.

**Table 1:** Sex distribution according to age group

**Age group**	**Male (N/%)**	**Female (N/%)**	**Total (N/%)**
<20	5 (3.5)	1 (0.7)	6 (4.2)
21-30	8 (5.6)	6 (4.2)	14 (9.8)
31-40	10 (7.1)	7 (4.9)	17 (12.0)
41-50	45 (31.7)	22 (15.5)	67 (47.2)
51-60	19 (13.4)	7 (4.9)	26 (18.3)
61-70	8 (5.6)	1 (0.7)	9 (6.3)
>70	3 (2.1)	0	3 (2.1)
Total	98 (69.0)	44 (31.0)	142 (100)

### Etiological risk factors

Seventy-four (52.1%) patients stated a history of ingestion of foods stored in humid conditions (a likely suspected source of aflatoxin B1 exposure). Eighty-five (59.9%) patients had a past history of jaundice and 32 (22.5%) had scarification marks. The use of traditional herbal concoctions was documented in 82 (57.7%) patients. A history of heavy alcohol consumption was reported in 86 (60.6%) patients. A past blood transfusion was documented in 28 (19.7%) patients.

### Clinicopathological presentation

The duration of symptoms before presentation ranged from 2 to 48 weeks with a median of 16 weeks and (IQR of 12 to 20 years). The majority of patients (125, 88.0%) had symptoms of more than 16 weeks duration at the time of presentation. Table 2 shows the distribution of patients according to clinical presentation.

**Table 2:** Distribution of patients according to clinical presentation

**Clinical presentation**	**Frequency**	**Percentages**
Right upper abdominal pain	138	97.2
Weight loss	132	92.9
Right hypochondrial swelling	116	81.7
Pallor	102	71.8
Jaundice	74	52.1
Itching	70	49.3
Ascites	68	47.9
Hematemesis	34	23.9
Hepatic bruit	18	12.7

A pre-existing medical illness was recorded in 9 (6.3%) patients; diabetes mellitus in three patients, hypertension in two patients, tuberculosis and chronic renal failure in two patients each respectively.

Thirteen (9.2%) patients were HIV positive. Of these, 3 (23.1%) patients were known cases on antiretroviral therapy (ARV) and the remaining 10 (76.9%) patients were newly diagnosed patients.

According to the Okuda classification [16], the majority of patients (76.8%) presented in stage III, while 23.2% of patients presented in stage II. No patients presented with stage I. Distant metastases were documented in 38 (26.8%) patients and occurred mainly to the lungs, bones, and skin. The majority of patients, 75 (52.8%) had both right and left lobe involvement. The left and right lobes were involved in 42 (29.6%) and 25 (17.6%) patients, respectively. The various histopathological grades of the cancer included welldifferentiated HCC (59.2%), anaplastic HCC (28.1%), and moderately differentiated HCC (12.7%). The histological types were trabecular and/or sinusoidal pattern (68 patients 47.9%), pseudoglandular and/or acinar pattern (35 patients 24.6%), compact pattern (25 patients 17.6%), clear cell (9 patients 6.3%), and scirrhous and/or fibrolamellar (5 patients 3.5%). The presence of cirrhosis coexisting with HCC was reported in 94 (66.2%) patients. The Child-Pugh classification of stage of the disease is as shown in Table 3.

**Table 3:** Distribution of patients according to demographic and clinicopathological characteristics

**Study variable**	**Number of patients (N)**	**Percentages (%)**
Age (in years)		
≤45	97	68.3
>45	45	31.7
Sex		
Male	98	69.0
Female	44	31.0
Duration of symptoms (in weeks)		
≤16	17	12.0
>16	125	88.0
HBsAg		
Positive	94	66.2
Negative	48	33.8
HCV antibody		
Positive	24	16.9
Negative	118	83.1
HIV status		
Positive	13	9.2
Negative	129	90.8
HCC type		
Multiple discrete lesions	70	49.3
Diffuse lesions	52	26.6
Solitary lesions	20	14.1
Solitary lesions		
>10 cm	12	60.0
5-10 cm	5	25.0
<5 cm	3	15.0
Okuda stage		
I	-	-
II	34	23.2
III	108	76.8
Child-Pugh class		
A	-	-
B	39	27.5
C	103	72.5
Histopathological grade		
Well differentiated	84	59.2
Moderately differentiated	18	12.7
Poorly differentiated	40	28.1

### Radiological and laboratory investigations

Abdominal ultrasonographic appearance performed in all the HCC patients revealed multiple discrete lesions (49.3%), diffuse lesions (26.6%), and solitary lesions (14.1%). The size of the single focal lesion was greater than 10 cm in 60.0% of patients, 5 to 10 cm in 25.0% of patients, and only 15.0% of patients had a lesion less than 5 cm.

Chest X-rays performed in 102 (71.8%) patients confirmed lung metastasis in 24 (23.5%) patients. No patient had a computed tomography (CT) scan or magnetic resonance imaging (MRI) examination due to the lack of these imaging facilities at our centre. Liver function tests were performed in all patients and revealed abnormal results in 134 (94.4%) patients. Out of 142 patients, 94 (66.2%) tested positive for HBsAg, while anti-HCV antibodies were detected in 24 (16.9%) patients. Eleven (7.7%) patients had dual infection. Thirteen (9.2%) patients tested positive for HIV infection. The CD4+ count among HIV-positive patients was available in only 8 patients and ranged from 122 to 788 cells/μl with a median of 256 cells/μl. A total of three HIV-positive patients (37.5%) had a CD4+ count below 200 cells/μl and the remaining five patients (62.5%) had a CD4+ count of ≥200 cells/μl.

### Treatment modalities

All of our patients (100%) received supportive therapy only because of the advanced nature of the disease and general deteriorated condition. No patients received any curative treatment such as liver transplantation, percutaneous ethanol ablation, or radio frequency ablation due to the lack of these treatment modalities in our setting and due the advanced nature of the disease at presentation.

### Outcome and follow-up of patients

Out of 142 patients, 92 developed complications giving a complication rate of 64.8%. Of these, coagulopathy was the most common complication, accounting for 45.7% of cases (Table 4).

**Table 4:** Distribution of patients according to the complications

**Complications**	**Frequency**	**Percentages**
Coagulopathy	42	45.7
Hepatic encephalopathy	38	41.3
Massive hemoperitonium	23	25.0
Upper gastrointestinal bleeding	14	15.2
Hepatorenal syndrome	8	8.7
Bacterial peritonitis	5	5.4

Sixty-six patients died in hospital, giving a mortality rate of 46.5%. The median duration from the onset of symptoms to time of death was 16 weeks (IQR of 12 to 20 weeks). According to multivariate logistic regression analysis, associated comorbidity, HIV positivity, CD4+ count <200 cells/μl, high histological grade and advanced stage of the tumor, presence of metastases at the time of diagnosis, and associated complications were the main predictors of death (*P* < 0.001). The causes of the deaths are shown in Table 5.

**Table 5:** Distribution of patients according to the causes of death (N = 66)

**Cause of death**	**Frequency**	**Percentage**
Hepatic failure	34	55.5
Intraperitoneal bleeding	18	27.3
Hemorrhagic shock from variceal bleeding	16	24.2
Intra-tumor hemorrhage	11	16.7
Associated HIV complications	9	13.6
No established cause	12	18.2

The length of hospital stay ranged from 6 to 45 days with a median of 14 days (IQR of 10 to 16 days).

Out of 142 patients, 68 (47.9%) were discharged aliveand a further 8 (5.6%) patients were discharged against medical advice.

Patients were followed up on for a period of 12 months. At the end of 12 months, only 22 (28.9%) patients (survivors) were available for follow-up and the remaining 54 (71.1%) patients were lost to follow-up.

## Discussion

Hepatocellular carcinoma is one of the most common cancers in the world and is associated with poor prognosis [11-13]. According to recent reports, the incidence of HCC has increased sharply in the last 5 to 10 years [17]. In this review, hepatocellular carcinoma accounted for 4.6% of all diagnosed malignancies seen during the study period in our setting. A high figure of 5.6% was also reported in one study in Turkey [18]. This may be related to differences in diagnostic approaches and levels of exposure to other carcinogenic or mutagenic environmental agents, such as aflatoxin B1.

In this study, HCC was more prevalent in males than in females, with a male to female ratio of 2.2:1. This is in agreement with other many studies [2,3,11-13,17,18]. The reasons for the male predominance may be explained in part by the differences in exposure to risk factors such as a higher prevalence of persistent HBV infection, alcohol abuse, groundnut chewing, and smoking in men than in women [19]. Genetic and hormonal factors may also be important [20-22]. It has been speculated that estrogens and androgens could modulate hepatocarcinogenesis and explain the higher incidence of HCC in men [23].

Studies in developing countries have shown that HCC tends to afflict people in their third and fourth decades of life, unlike in developed countries where the disease is prevalent in the older population [17]. In agreement with other studies [11,12,17,18,24], the peak age of incidence of hepatocellular carcinoma in our study was found to be in the fourth decade of life, which is about a decade or two earlier compared to the findings in developed countries. The occurrence of HCC in a younger age group compared to the Western population suggests an early exposure to carcinogenic factors in our patients. We noted a tendency for patients with HBV-associated HCC to present at a younger age than those with HCV-associated HCC, which is in line with previous observations [25]. We could not establish the reason for this occurrence.

Hepatocellular carcinoma has been reported in most studies to be more prevalent in people with low socioeconomic status [2,3,13]. This finding is reflected in our study in which the majority of patients were farmers coming from rural areas located a considerable distance from the study area and more than 80% of them were unemployed. Similar observations were reported by other studies in developing countries [2,3,13,17,26]. This observation has an implication on the accessibility of healthcare facilities and awareness of the disease.

The major risk factors for developing hepatocellular carcinoma vary by region and degree of national development [26,27]. In the present study, HBV infection was the most common etiological factor in our environment as more than 65% of patients tested positive for HBsAg, which is consistent with the findings of other studies [2,3,11-13,18,26,27]. On other hand, only 16.9% of the patients were positive for the HCV antibody which indicates that HCV is not as important as HBV in the etiology of HCC in this environment. This is contrary to what occurs in the United States, Europe, and Japan where 50 to 75% of patients with HCC had evidence of HCV infection [28-30]. The high HBV seroprevalence among patients with HCC in our setting calls for including HBV vaccination in the national program on immunization in Tanzania.

In this study, HBsAg were negative in 33.8% of the HCC patients suggesting exposure to other strong etiological factors such as aflatoxins and alcohol abuse. Exposure to alcohol abuse was particularly high in this study (60.6%). Possible exposure to aflatoxin B1, though not proven as no test was conducted to incriminate it, is highly suspected as a causative factor in this study as 52.1% of the patients were exposed to a likely source of aflatoxin B1 contamination (inappropriately preserved food). The duration of exposure to this could not be ascertained. The ingestion of herbal preparations for various ailments is widespread in Tanzania and more than half of the patients in this study abused various herbal preparations. Furthermore, the use of medicinal herbs was reported as an important co-factor with aflatoxin in the development of HCC in Ethiopia [31].

The prevalence of HIV infection among patients with HCC in the present study was 9.2% which is higher than the 6.5% [32] in the general population in Tanzania. However, the overall HIV seroprevalence in this study may actually be an underestimate and the magnitude of the problem may not be apparent because many patients were excluded from the study due to a failure to meet the inclusion criteria. We could not establish the reason for the high seroprevalence of HIV among these patients although it is possible that these patients have an increased risk of exposure to HIV infection. This calls for further research on this observation. HIV infection was found to be associated with poor outcome and this finding call for routine HIV screening in patients suspected to have HCC.

The clinical presentation of hepatocellular carcinoma in our patients is not different from those in other studies [3,11,12], with right hypochondrial pain, weight loss, and abdominal swelling being the commonest symptoms with which our patients presented. Hepatic bruit over an enlarged liver is considered a reliable diagnostic sign. However, this sign was elicited in only 12.7% of the patients which is similar to what was reported by Ndububa *et al.*[11] in Nigeria. This presentation generally reflects the problem of late presentation that is common in Tanzanian patients.

The majority of patients in this study presented late with an advanced stage of the disease which is in keeping with other studies performed in developing countries [2,3,11-13,18,26,27]. The late presentation in our study may be attributed to lack of awareness of the disease, a lack of accessibility to healthcare facilities, and a lack of advanced diagnostic investigations such as CT and MRI scans in this region. The late presentation of cases is an area of cancer care in our center that requires urgent attention. Detecting primary cancer at an early stage contributes to improved chances for successful treatment and thus for survival.

Distant metastases were documented in 26.8% of the patients and occurred mainly to the lungs, bones, and skin. This figure is higher than that reported in other reports [3,11,12,26]. The high rate of distant metastases in this study is attributed to the late presentation in the majority of patients and this confirmed the highly metastatic potential of HCC.

It was found in this study that the majority of patients (52.8%) had both right and left lobe involvement, whereas the left and right lobes were involved in 29.6% and 17.6% of the patients respectively. This was contrary to the findings in the study of El-Zayadi *et al.*[17] who found 65% affecting the right lobe, 13.4% the left lobe, and 21.6% affecting both lobes. We could not establish the reason for this anatomical distribution. Histopathologically, the trabecular type was the most commonly encountered variety. This finding is similar to that noted by most other researchers; the trabecular type being reported to be the most common type and constituting 60 to 75% of HCC [33].

The presence of cirrhosis coexisting with HCC was reported in 66.2% of the patients, which is in agreement with the figure of 60 to 100% reported in literature [16]. Globally, more than 80% of HCC cases occur in individuals with cirrhosis of the liver [34], and the annual incidence of HCC in cirrhotic patients is 1 to 6% [18,35]. This suggests that cirrhosis is the main risk factor for this tumor.

The ultrasonographic appearance of HCC performed in all patients concurs with what was described by Nwokediuko *et al.*[36] in Nigeria. Multinodular HCC is known to be more prevalent in patients with multiple risk factors for this cancer [37]. This suggests that the risk factors for most of our patients with HCC may be multiple.

The treatment of HCC requires a multidisciplinary approach. Treatment modalities of HCC include surgery in the form of either resection or transplantation, locoregional therapy including transarterial chemoembolization, percutaneous ethanol injection, or radiofrequency ablation. These techniques may be curative in patients with early stage of the disease. However, these techniques have yet to be used on a large scale in Sub-Saharan Africa, including Tanzania, and no reports of the results obtained have been published. The treatment of HCC in resource-limited countries like ours has been and remains largely unrewarding. Late presentation and the advanced stage of the disease at the time of diagnosis, as well as inadequate diagnostic and treatment facilities for the tumor pose enormous challenges in managing HCC in these countries [13]. Surgery in the form of either resection or transplantation is currently the only golden standard treatment for HCC with curative potential [3,13]. However, curative surgery in most developing countries is limited because most of the patients we see in our environment present late with an advanced stage of the disease at the time of diagnosis, for which only palliative treatment is possible. This fact is reflected in our study as all of our patients received supportive therapy only because of the advanced nature of the disease and general deteriorated condition. Late presentation still poses a challenge to the successful management of HCC in our setting and requires public education.

As reported by Mustapha and Pindiga [12] in Nigeria, coagulopathy was the most common complication in this study, occurring in 45.7% of the patients. Therefore, it is important to do the clotting profile of all HCC patients, especially if needle biopsy of the liver is being contemplated, in order to ovoid continuous bleeding from the biopsy site.

The overall mortality rate in this study was 46.5%, a figure which is higher than that reported by Salem *et al.*[26] in Yemen. The mean duration from onset of symptoms to time of death was 16 weeks, which is in agreement with the findings from other studies [11,12,38]. Hepatic failure and intraperitoneal hemorrhage were the most common causes of death. In this study, the mortality rate was significantly high in patients with associated comorbidity, HIV positivity, CD4+ count <200 cells/μl, high histological grade and advanced stage of the tumor, presence of metastases at the time of diagnosis, and associated complications. Addressing these factors responsible for high mortality in our patients is mandatory to be able to reduce mortality associated with this disease.

The overall median duration of hospital stay in the present study was 14 days which is higher than that reported by Ajayi *et al.*[39] in Nigeria. However, due to the poor socioeconomic conditions in Tanzania, the duration of inpatient stay for our patients may be longer than expected.

In keeping with other studies [11,12], the outcome of patients in the present study was generally poor, as less than 50% of patients were discharged alive. This is not surprising because the prognosis of HCC all over the world is generally poor. The reasons for the poor outcome in our HCC patients may be attributable to late presentation so that the majority of patients had an advanced stage of the disease at presentation.

Self-discharge by the patient against medical advice is a recognized problem in our setting. In this study, 5.6% of patients were discharged against medical advice. A high figure of 9.4% was reported by Ajayi *et al.*[39] inNigeria. We could not establish the reasons for this difference.

The follow-up of patients in this study was generally poor as more than 70% of patients were lost to follow-up at the end of the follow-up period. This observation concurs with other studies performed in developing countries [2,11,13,38]. Poor follow-up of patients in our study remain a cause for concern. The loss to follow-up after hospital discharge may be the result of poverty, the long distance from the hospital centers, and ignorance.

The potential limitation of this study is the fact that information about some patients was incomplete in view of the retrospective nature of the study and this might have introduced some bias in our findings. However, despite this limitation, the study has provided local data that can help healthcare providers in the management of patients with HCC. The challenges identified in the management of HCC in our setting need to be addressed in order to deliver optimal care for these patients.

## Conclusions

Hepatocellular carcinoma patients in this region are relatively young at diagnosis and the majority of them present late with an advanced stage of the disease and a high rate of distant metastasis. Hepatitis B virus infection and alcohol abuse are the most common etiological factors in our environment. Lack of awareness of the disease, poor accessibility to healthcare facilities, lack of diagnostic facilities, the high cost of care, and a high morbidity and mortality rate are among the hallmarks of the disease in this region and pose a great challenge in the management of these patients. Therefore public enlightenment, early diagnosis, and effective cost-effective treatment and follow-up will help reverse this trend. In addition, early HBV immunization of all children will go a long way towards reducing the incidence of HCC in later life.

## Abbreviations

HCC: Hepatocellular carcinoma; HBC: Hepatitis B virus; AFP: Alpha fetoprotein; HBsAg: Hepatitis B surface antigen.

## Competing interests

The authors declare that they have no competing interests.

## Authors’ contributions

HJ and PLC participated in study design, literature search, data analysis, manuscript writing and editing. In addition, PLC participated in submission of the manuscript. SEM, PFR, NM and SEK participated in data analysis, manuscript writing and editing. In addition, SEK supervised the study. All the authors read and approved the final manuscript.
